# Microclimate and the vertical stratification of potential bridge vectors of mosquito‑borne viruses captured by nets and ovitraps in a central Amazonian forest bordering Manaus, Brazil

**DOI:** 10.1038/s41598-021-00514-0

**Published:** 2021-10-26

**Authors:** Adam Hendy, Danielle Valério, Nelson Ferreira Fé, Eduardo Hernandez-Acosta, Claudia Mendonça, Eloane Andrade, Igor Pedrosa, Edson Rodrigues Costa, José Tenaçol Andes Júnior, Flamarion Prado Assunção, Bárbara Aparecida Chaves, Vera Margarete Scarpassa, Marcelo Gordo, Michaela Buenemann, Marcus Vinícius Guimarães de Lacerda, Kathryn A. Hanley, Nikos Vasilakis

**Affiliations:** 1 Department of Pathology, Sealy Center for Vector-Borne and Zoonotic Diseases, Center for Biodefense and Emerging Infectious Diseases, Center for Tropical Diseases, Institute for Human Infections and Immunity, University of Texas Medical Branch, Galveston, TX USA; 2 Fundação de Medicina Tropical Doutor Heitor Vieira Dourado (FMT-HVD), Manaus, Amazonas Brazil; 3 Department of Biology, New Mexico State University, Las Cruces, NM USA; 4 Laboratório de Biologia da Conservação, Projeto Sauim-de-Coleira, Instituto de Ciências Biológicas, Universidade Federal Do Amazonas, Manaus, Amazonas Brazil; 5 Coordenação de Biodiversidade, Instituto Nacional de Pesquisas da Amazônia, Manaus, Amazonas Brazil; 6 Department of Geography, New Mexico State University, Las Cruces, NM USA; 7 Instituto Leônidas & Maria Deane (Fiocruz - Amazônia), Manaus, Amazonas Brazil

**Keywords:** Ecological epidemiology, Viral infection, Entomology

## Abstract

In the Americas, some mosquito-borne viruses such as Zika, chikungunya, and dengue circulate among humans in urban transmission cycles, while others, including yellow fever and Mayaro, circulate among monkeys in sylvatic cycles. The intersection of humans and wildlife at forest edges creates risk for zoonotic virus exchange. We built a scaffold tower at the edge of a treefall gap in rainforest bordering Manaus, Brazil, to identify vectors that may bridge transmission between humans and monkeys. We vertically sampled diurnally active, anthropophilic mosquitoes using handheld nets at 0, 5, and 9 m and container-breeding mosquitoes in ovitraps at 0, 5, 10, and 15 m. *Haemagogus janthinomys* and *Psorophora amazonica* were present in high relative abundance in nets at each height sampled, while anthropophilic species were uncommon in ovitraps. *Hg. janthinomys* was more abundant at elevated heights than at ground level, while *Ps. amazonica* abundance was not significantly stratified across heights. The presence of each species increased with increasing 7-day rainfall lagged at 1 week, and at 1 and 4 weeks prior to collection, respectively. In addition, *Hg. janthinomys* was most frequently collected at 29.9 °C, irrespective of height. These data provide insight into the potential role of each species as bridge vectors.

## Introduction

Arboreal mosquitoes transmit sylvatic arthropod-borne viruses (arboviruses), such as yellow fever virus (YFV, *Flaviviridae*: *Flavivirus*), among wildlife in forests in Africa and Latin America^1^. YFV was first introduced to the Americas in the seventeenth century^2^, transported by *Aedes aegypti* mosquitoes among shipments of enslaved people from West Africa^3^. Having arrived in the neotropics, the virus caused devastating urban outbreaks and eventually spilled back into a sylvatic cycle involving susceptible monkeys and *Haemagogus* and *Sabethes* species mosquitoes^1,2^. Beginning in the 1940s, campaigns to eliminate *Ae*. *aegypti* throughout much of Latin America extinguished urban YFV transmission in the region^1,3^. Human disease is now a consequence of spillover from the sylvatic cycle^4^ mediated by mosquito bridge vectors that bite both humans and monkeys where they occur in close contact, such as at forest edges^5^. The discontinuation of *Ae. aegypti* control programs in the 1970s has allowed this vector to re-establish across the neotropics. Despite this, YFV was largely confined to the Amazon basin until the early 2000s, when it resurfaced in several southeastern states including Minas Gerais, São Paulo, and Rio Grande do Sul^6^. These outbreaks were followed by the largest modern epidemic of YFV in Brazil^7^, which between 2016 and 2019, resulted in 2,166 confirmed human cases and 751 deaths caused by spillover from the sylvatic cycle^3^. In addition, at least 1,567 YF infections were detected in monkeys during this period, of which 90.9% occurred in southeastern Brazil^8^. While there was no clear evidence of *Aedes*-borne urban transmission among humans^7,9^, the virus threatened large, immunologically naïve, urban populations in the states of Rio de Janeiro and São Paulo^4,9,10^ with the potential for devastating consequences.

In the presence of susceptible hosts and competent vectors, areas at risk of arbovirus spillover are also likely to be at risk of spillback from humans to wildlife. In Latin America, dengue (DENV, *Flaviviridae*: *Flavivirus*), chikungunya (CHIKV, *Togaviridae*: *Alphavirus*), and Zika (ZIKV, *Flaviviridae*: *Flavivirus*) viruses have all emerged or resurged in recent years, where they currently circulate in urban cycles maintained by *Ae. aegypti* and *Ae. Albopictus*^11,12^. There is now concern that one or more of these viruses may spill back into neotropical forests in the region, which would make their elimination almost impossible and thereby create an enduring threat to human health^11,13,14^.

Which mosquito species effectively bridge urban and sylvatic cycles is determined in part by their vertical stratification relative to humans living at ground level and sylvatic hosts which may prefer different heights within the forest canopy. We recently identified *Haemagogus janthinomys*, *Sabethes chloropterus*, and *Psorophora amazonica* as potential bridge vectors of mosquito-borne arboviruses in a rainforest reserve bordering the city of Manaus, Brazil^15^. The two former species are major sylvatic vectors of YFV in Latin America and are commonly active in forest canopies^16^, a behavior termed acrodendrophily^17^. The latter is a poorly studied species of unknown vector status^15^. All three species opportunistically feed on humans and are primarily active during the daytime^15,16^. Twenty-four-hour BG-Sentinel trap collections made at 0, 5, 10, and 15 m in height revealed that *Hg. janthinomys* and *Sa. chloropterus* were significantly stratified and occurred most frequently above the ground, while *Ps. amazonica* was the most abundant species at each height sampled and showed no significant stratification^15^. We also found positive associations between 7-day cumulative rainfall in the weeks prior to collections and the occurrence of key taxa. *Haemagogus* species and *Sa. chloropterus* were associated with rainfall at lags of one and three weeks, respectively, while *Ps. amazonica* was associated with rainfall at lags of one and four weeks. In addition, analyses of relationships between microclimate and the occurrence of *Haemagogus* and *Sabethes* mosquitoes showed that detection of both genera significantly increased with increasing temperature and decreasing relative humidity at several heights sampled^15^. These findings support anecdotal evidence that *Hg. janthinomys*^18^ and other acrodendrophilic species^16^ increase in abundance at ground level at forest edges, where high temperatures and low humidity create conditions similar to those found in the forest canopy^5,15^.

An important limitation of the above study was our reliance upon BG-Sentinel traps for sampling. These traps were designed for the collection of diurnally active urban *Aedes* vectors^19^, and although they should provide reliable estimates of the relative stratification of *Hg. janthinomys*, *Sa. chloropterus*, and *Ps. amazonica*, they do not permit inferences to be made about the absolute abundance of the anthropophilic fraction of these species. Indeed, based on these collections, both *Hg. janthinomys* and *Sa. chloropterus* appeared to be relatively scarce within the mosquito community, but all methods of sampling are biased towards the collection of certain species, or developmental and physiological stages of mosquito populations^20–25^. In addition, spatial bias, caused by the type of habitat being sampled^26^ or by the positioning of traps relative to the forest canopy^15^ and forest edges^5^, may influence the composition of species collected, while temporal bias caused by the season^27^ or year of collection^28^ may also have an effect. Thus, robust inferences about highly diverse mosquito communities require a diversity of sampling approaches deployed across space and time.

To meet this need, we sampled diurnally active, anthropophilic mosquitoes, the group most likely to effect spillover and spillback, using handheld nets, and ovipositing mosquitoes using artificial and natural containers. Sampling took place at a 10 m scaffold tower constructed at the edge of a treefall gap in the same rainforest reserve that we initially surveyed using BG-Sentinel traps. Focusing on potential arbovirus bridge vectors, we hypothesized that results from net collections would corroborate our initial conclusions regarding stratification and relationships with microclimate but would reveal different patterns in the relative abundance of key species.

## Methods

### Study area

**Figure 1:**
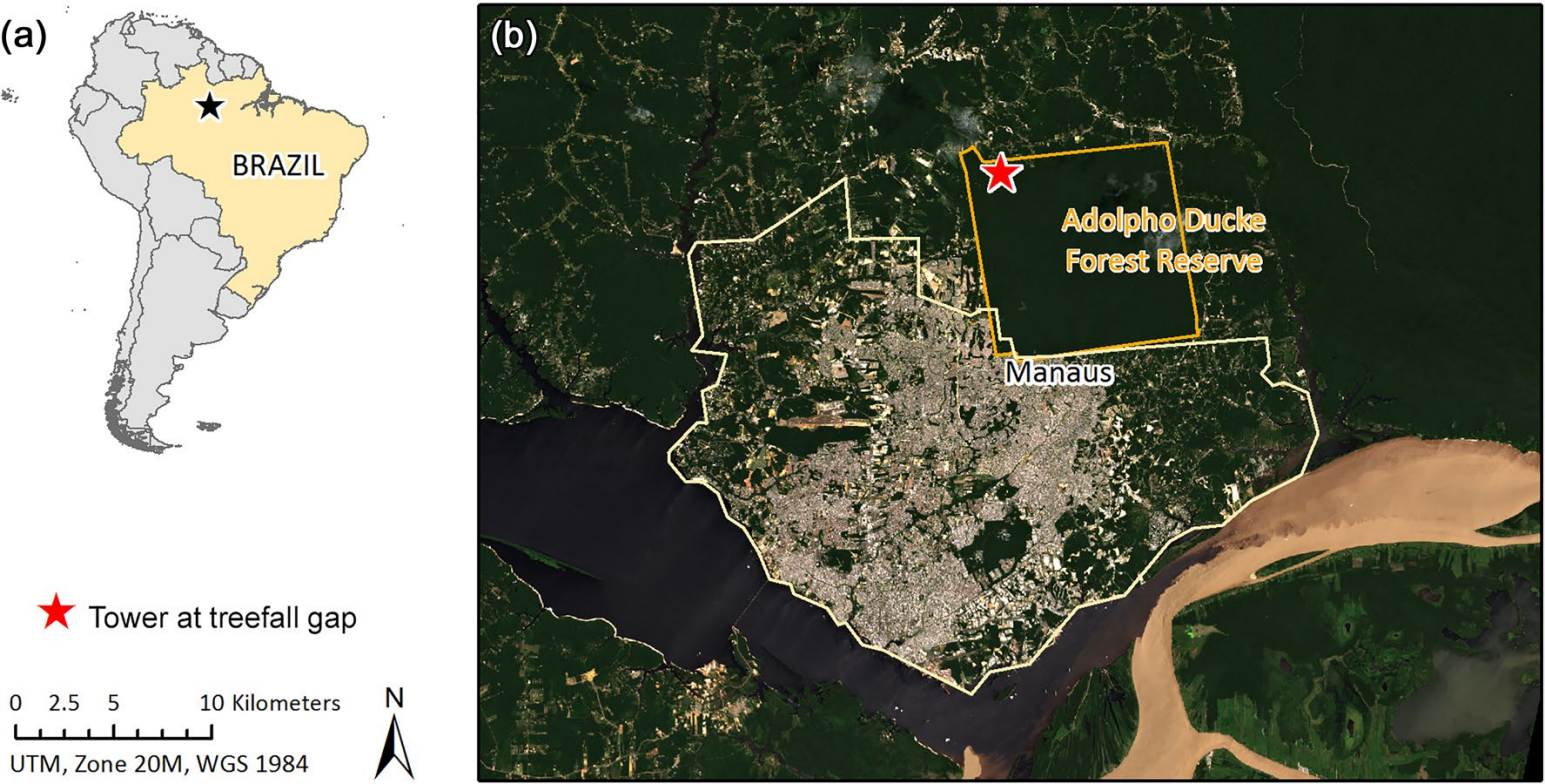
Study site at the Adolpho Ducke forest reserve bordering Manaus. (**a**) shows the location of Manaus in Brazil (black star) and (**b**) the location of the tower and treefall gap (red star). The orange border shows the approximate extent of the reserve. Map created using ArcGIS Desktop 10.7.1 (ESRI, Redlands, California).

The study was conducted at the Adolpho Ducke forest reserve (Ducke) bordering Manaus in Amazonas State, Brazil (Fig. 1). Locally circulating urban arboviruses include DENV, CHIKV, and ZIKV^29^, while YFV is present in a sylvatic cycle^30^. The reserve comprises 100 km^2^ of primary rainforest which borders the city and is contiguous with the surrounding Amazon^31^. Humans, wildlife, and both urban and sylvatic mosquitoes exist in close contact, creating conditions that are favorable to arbovirus spillover and spillback. Ducke is mainly accessed by researchers and students and is home to six monkey species^31^. Several are common to the forest understory and edge, namely the golden-faced saki (*Pithecia chrysocephala*), the pied tamarin (*Saguinus bicolor*), and the tufted capuchin (*Sapajus apella*)^32–34^, while the Guianan red howler monkey (*Alouatta macconnelli*), Guiana spider monkey (*Ateles paniscus*) and bearded saki (*Chiropotes chiropotes*) are also present^15^. The rainy season generally lasts from November to May and the dry season lasts from June to October^31^.

### Study site

The study site, referred to as site B in our previous publication^15^, was established at a large treefall gap 480 m from the forest edge in the northwestern corner of the reserve (02.92537° S, 059.96582° W). The surrounding forest was populated by trees 20 – 30 m in height and mosquitoes present in the area included *Hg. janthinomys*, *Sa. chloropterus*, and *Ps. amazonica*.

### Adult mosquito collections

**Figure 2:**
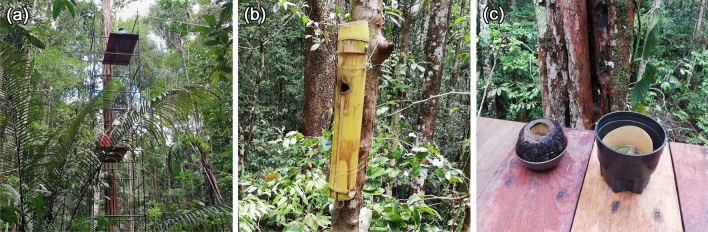
Study site at the edge of a treefall gap. (**a**) shows the scaffold tower with platforms at 5 m and 9 m, (**b**) the bamboo ovitrap with a 2 cm lateral hole attached to a tree next to the 5 m platform, and (**c**) the fruit pod and standard ovitraps lined with germination paper on the 9 m platform.

A 10 m tall scaffold tower (Fig. 2a), with platforms constructed at 5 m and 9 m, was built next to a standing tree (*Geissospermum argenteum*) at the edge of the treefall gap to investigate the stratification of diurnally active, anthropophilic mosquito species (Supplementary Data S1). Mosquitoes were collected between 10:00 and 15:00 on each sampling day, a time period during which the species of interest tend to be active^35^. Collections were made by three people using 30 cm diameter handheld nets who worked simultaneously at heights of 0, 5, and 9 m. Each mosquito was placed individually in a numbered 3.5 mL push cap tube (Sarstedt AG & Co., Nümbrecht, Germany) and the time of collection was recorded to the nearest minute. Collections were restricted to a maximum of one mosquito per minute due to the number of tubes available. Ten collectors were involved in sampling over the course of the study. The height at which a collector worked was rotated daily where possible to minimize potential effects of collector bias. Collections were generally made twice a week for a total of 36 days between 13 November 2019 and 9 April 2020, when sampling was terminated due to the COVID-19 pandemic. Mosquitoes were stored in the field on dry ice and were transferred to a −80 °C freezer at the Fundação de Medicina Tropical Doutor Heitor Vieira Dourado (FMT-HVD) until identified.

Hygrochron iButton data loggers (Maxim Integrated, San Jose, California), placed in nylon mesh bags and attached to the tower at 0, 5, and 9 m, were used to record temperature (°C) and relative humidity (%) at 30-min intervals (on the hour and at 30 min past each hour) during each sampling period. Each individual mosquito was assigned to the temperature and humidity datapoint closest to its time of collection, with mosquitoes collected at 15 or 45 min past each hour assigned to the following datapoint. Precipitation data, obtained from the automated INMET meteorological station (Code: Manaus-A101, OMM: 81730) located within Manaus (3.103682° S, 60.015461° W), were used to calculate cumulative 7-day rainfall at lags of 1, 2, 3, and 4 weeks prior to each collection to test associations between past rainfall and the occurrence of adult mosquitoes.

### Ovitrap collections

The stratification and trap preference of ovipositing mosquitoes was investigated by collecting mosquito eggs in three types of water-holding container (Figs. 2b, c, Supplementary Data S2). These were standard black plastic ovitraps (Geronimo Strategic, El Paso, Texas), Brazil nut tree (*Bertholletia excelsa*) fruit pods, and 30 cm sections of bamboo with a lid and 2 cm diameter lateral hole. One of each container type was placed at each of four heights (0, 5, 9, and 15 m) in a factorial blocked design. The containers at 0, 5, and 9 m were placed on the tower, while those at 15 m were suspended on a rope hanging from a branch above the tower. Each container was lined with seed germination paper (Nasco, Fort Atkinson, Wisconsin) as an oviposition substrate, along with several decaying leaves and water from a nearby stream. Containers were left in the field for seven days before the water and germination paper were collected and replaced. Ten collections were made between 17 January and 9 April 2020.

Eggs were reared to adults at ambient temperatures (26 °C ± 1) and relative humidity (70–80%) in an insectary at FMT-HVD. Germination papers and water from each container were placed in rearing trays and topped-up with distilled water if necessary. If eggs did not hatch after three days, papers were dried for three days and then resubmerged, up to a maximum four times. Larvae were fed with fish food (Oscar Gold Ornamental Fish Food, Hai Feng, Taiwan) as required. Pupae were separated and placed in cups of distilled water in cages containing cotton wool soaked with 10% glucose solution. Adults were kept alive for 24 h after eclosion to allow male genitalia to rotate before being stored at -20 °C until identified. Any larvae remaining in trays one month after rearing were discarded.

### Mosquito identifications

Mosquitoes were placed on a chill table (BioQuip, Rancho Dominguez, California, USA) and identified to species using a stereomicroscope and taxonomic keys as previously described^5,15^. Genus and species names follow the Walter Reed Biosystematics nomenclature^36^. Samples were stored at −80 °C for future arbovirus screening.

### Statistical analyses

Where possible, analyses were performed that enabled comparisons with our previous work using BG-Sentinel traps^15^. Two approaches were used to investigate changes in the community composition of diurnally active, anthropophilic mosquitoes at each height. The first was the Morisita overlap index^37^ calculated using the PAST software package^38^. This is a commonly used measure of dispersion which provides a value between 0 (indicating no similarity between sample units) and 1 (complete similarity). The second approach was a principal components analysis of the relative frequency of each species followed by hierarchical clustering of communities. Contingency tables and Pearson’s chi-squared tests were used to compare the relative abundance of genera and species collected using nets and BG-Sentinel traps; rare genera were grouped and 1 count was added to each cell in the analysis to avoid zero values. Kruskal–Wallis tests followed by post-hoc Wilcoxon Each Pair tests were used to test the effect of height on the abundance of target species, and lag to first approach. Linear regressions were also used to test the effect of mosquito abundance, height, and mean daytime temperature on lag to first approach for *Hg. janthinomys*. Nominal logistic regressions were used to test the effect of date, height, mean daytime temperature, and 7-day cumulative rainfall at lags of 1, 2, 3, and 4 weeks on the occurrence (presence or absence) of target species. Nominal regressions were also used to investigate relationships between temperature, which was inversely correlated with relative humidity, and occurrence of these species.

For ovitrap collections, the Morisita overlap index was used to compare mosquito community composition between container types and among heights. Target species in ovitraps were insufficiently abundant for analysis of stratification in this study. All analyses were performed using JMP 14^39^ unless stated.

### Ethics and permits

We note that our method of collection differed from a human landing collection in that we measured mosquito approach without permitting mosquito landing. No skin was deliberately exposed to attract mosquitoes, and mosquitoes were netted prior to landing on a collector. All collectors are listed among the coauthors and were fully aware of the nature of the research. They wore trousers and either a long-sleeved shirt or repellent to minimize the risk of being bitten and had been vaccinated against yellow fever. Mosquito collections at the Ducke reserve were approved by local environmental authorities (SISBIO license 57003-6) and the study did not involve endangered or protected species.

## Results

### Variation in microclimate

**Figure 3:**
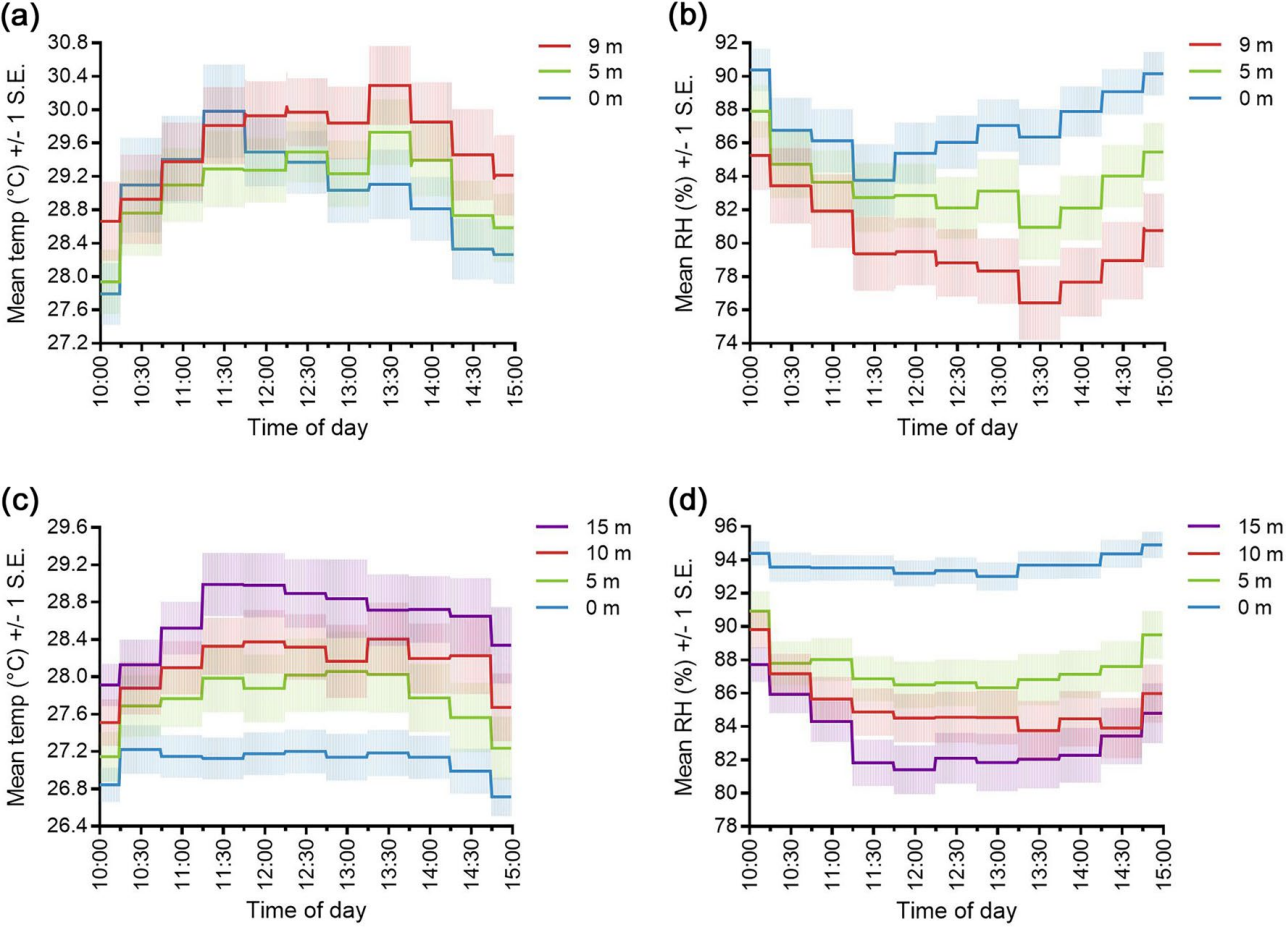
Variation in microclimate by height and collection method. (**a**) and (**b**) show the mean temperature (temp,°C) + / − 1 standard error (S.E.) and relative humidity (RH, %) + / − 1 standard error (S.E.) for net collections made at the tower between 10:00 and 15:00 in this study. (**c**) and (**d**) show corresponding data extracted from the BG-Sentinel trap study^15^.

Microclimate at the tower varied across the daily sampling period with temperatures highest and relative humidity lowest around midday and the early afternoon hours, although the time of peak temperature and nadir humidity varied by height (Fig. 3a, b). Mean temperature was highest at ground level at 11:30 (30.0 °C) when it was on average 0.2 °C hotter than at 9 m, whereas at 5 m and 9 m, it was highest at 13:30 (29.7 °C and 30.3 °C, respectively). The inverse was true for mean relative humidity, which was lowest at ground level at 11:30 (83.8%) and lowest at 5 m and 9 m at 13:30 (80.1% and 76.4%, respectively). Both variables showed substantial overlap in means and standard errors across the sampled heights during the morning hours, before diverging in the afternoon. For comparison, we extracted microclimate data from the corresponding sampling period in the BG-Sentinel trap study^15^, which revealed clear differences in temperature and humidity at each height sampled (Fig. 3c, d). BG-Sentinel traps were often hung beneath the forest canopy where it was considerably cooler and more humid than at the treefall gap, particularly at ground level.

### Community composition of diurnally active, anthropophilic mosquitoes

**Figure 4:**
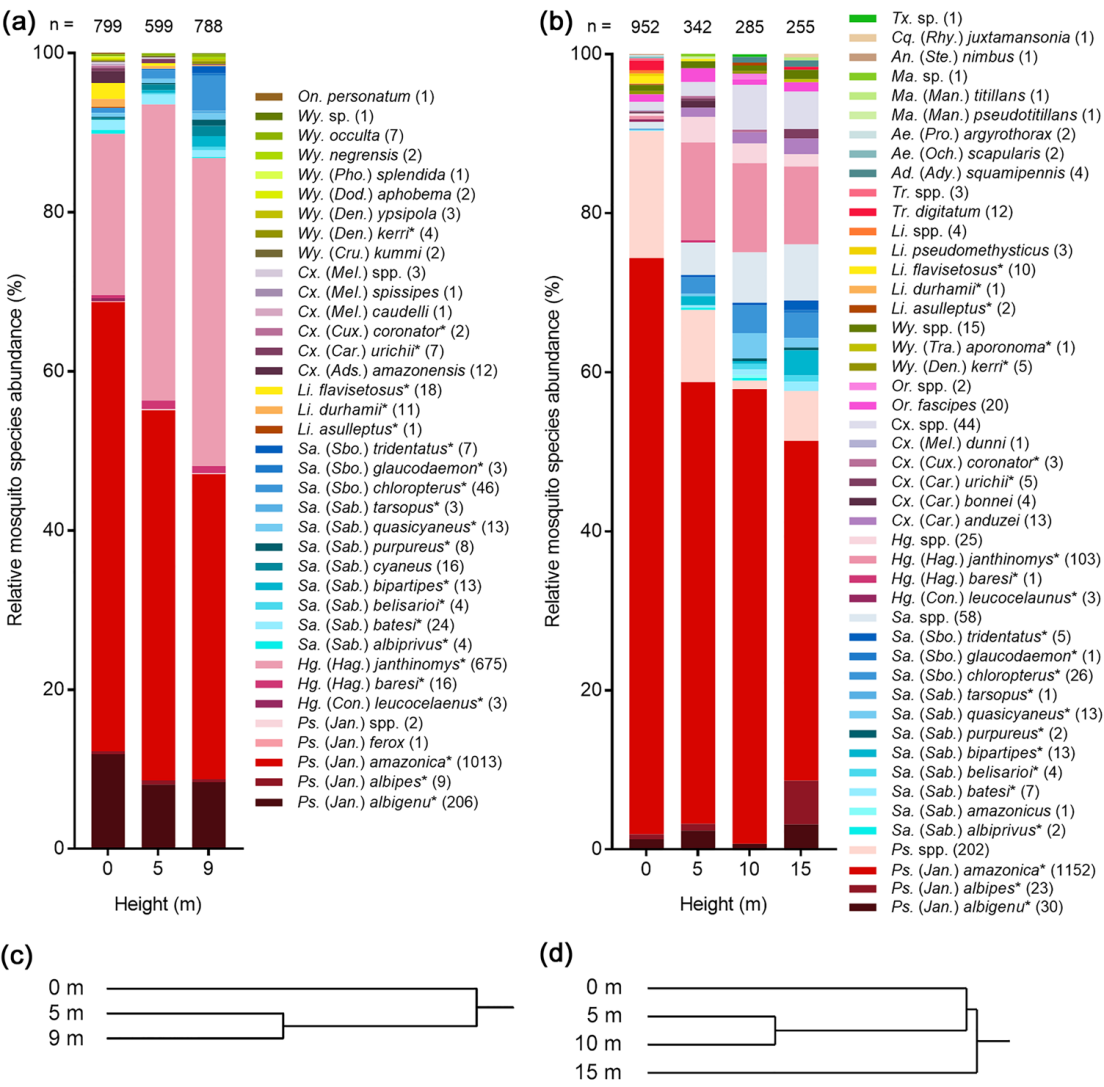
Relative mosquito species abundance (%) by height and collection method for net and BG-Sentinel trap collections. (**a**) shows the relative abundance of designated species collected using handheld nets, and (**b**) the relative abundance of designated species previously collected using BG-Sentinel traps^15^. Number of mosquitoes (n =) collected at each height listed at top of bar; number of individuals per species included in parentheses next to species name; *asterisk denotes species collected using both nets and BG-Sentinel traps; sp. = single species, spp. = potentially multiple species; genus and subgenus abbreviations follow Walter Reed Biosystematics Unit nomenclature^36^. (**c**) and (**d**) each show a dendrogram of PC1 from a principal components analysis of relative species frequency at each height sampled for respective species stacks. Panels (**b**) and (**d**) are reproduced from our previous study^15^ and reformatted for the current publication under a Creative Commons Attribution 4.0 International License (https://creativecommons.org/licenses/by/4.0/).

A total of 2146 adult mosquitoes representing seven genera and 34 species were collected using nets (Fig. 4a), of which 99.8% (2142/2146) were female and 99.7% (2140/2146) were identified to species level. Mosquito abundance was similar at ground level and 9 m but was slightly lower at 5 m, while species richness was higher at ground level (28 species), than at 5 m (18 species) and 9 m (22 species). *Psorophora* was the most abundant genus (1231 mosquitoes, 57.4% of the total catch), followed by *Haemagogus* (32.3%), and *Sabethes* (6.6%). The genera *Limatus* (1.4%), *Culex* (1.2%), *Wyeomyia* (1.0%), and *Onirion* (< 0.1%), formed just 3.7% of the total catch. In agreement with our BG-Sentinel trap collections from the same area (Fig. 4b), *Ps. amazonica* (82.4% of identified *Psorophora*) and *Hg. janthinomys* (97.3% of identified *Haemagogus*) dominated collections within their respective genera, while *Ps. albigenu* (16.8% of identified *Psorophora*) was also relatively abundant. *Sabethes* was the most diverse genus with 11 species collected, and *Sa. chloropterus* (32.4% of identified *Sabethes*) was the most abundant of these.

The Morisita overlap index for specimens identified to species level (n = 2140 mosquitoes) showed substantial overlap in community composition between the sampled heights. However, elevated (5 m and 9 m) communities were more similar to each other (0.966) than they were to the ground level community (0.945 and 0.896 for 5 m and 9 m comparisons with ground level, respectively). These results were consistent with clustering based on principal components generated using the summed numbers for each species per height (Fig. 4c) and agreed with the results of our BG-Sentinel trap collections (Fig. 4d).

### Comparing nets with BG-sentinel traps

When comparing handheld net collections in this study with those made using BG-Sentinel traps in the same area of the reserve^15^, there was a significant difference in relative genus abundance, pooled across height (Pearson’s chi-squared test, DF = 8, χ^2^ = 423.9, *P* < 0.0001) (Fig. 4). The relative abundance of *Haemagogus* was higher in nets than in traps, while for *Psorophora*, the reverse was true. The relative abundance of *Sabethes* was similar using both methods. When comparing the methods for individual species stratified by height, there was a significant difference in the relative abundance of *Hg. janthinomys* (DF = 2, χ^2^ = 22.7, *P* < 0.0001) which was collected in higher numbers at ground level compared with elevated heights in nets than in traps. There was also a significant difference in the relative abundance of *Ps. amazonica* (DF = 2, χ^2^ = 102.1, *P* < 0.0001), which was collected in lower numbers at ground level compared with elevated heights in nets than in traps. Small sample sizes precluded analysis of *Sabethes* mosquitoes at species level.

### Species abundance by height

As expected, *Hg. janthinomys* showed significant stratification in abundance across the sampled heights when analyzed using data summarized by date and height (Kruskal–Wallis test, DF = 2, χ^2^ = 10.05, *P* = 0.007). Post-hoc Wilcoxon Each Pair tests revealed this species to be more abundant at 9 m than at 0 m (*P* = 0.01), but there was no difference in abundance between 0 and 5 m or 5 m and 9 m (*P* > 0.09 for both comparisons). In contrast, there was no significant stratification in abundance of *Ps. amazonica* or *Ps. albigenu* across sampled heights (*P* > 0.12 for both comparisons). Data were further summarized by morning (10:00–11:59) and afternoon (12:00–14:59) due to the differences in stratification of microclimate during these periods (Fig. 3a, b). This showed that *Hg. janthinomys* abundance was not significantly stratified in the morning (Kruskal–Wallis test, DF = 2, χ^2^ = 4.7, *P* = 0.1) when microclimate generally overlapped across heights, but it was in the afternoon as microclimate became more distinct (Kruskal–Wallis test, DF = 2, χ^2^ = 11.4, *P* = 0.003). Again, post-hoc Wilcoxon Each Pair tests revealed *Hg. janthinomys* to be more abundant at 9 m than 0 m (*P* = 0.001) in the afternoon, whereas comparisons between 0 and 5 m (*P* = 0.085) and 5 m and 9 m (*P* = 0.051) were not significant. Neither *Ps. amazonica* or *Ps. albigenu* showed significant stratification in the morning or afternoon sampling periods (*P* > 0.08 for all comparisons). *Sabethes* species were not included in analyses of abundance due to small sample sizes.

### Lag to first approach

Lag to first approach was defined as the number of minutes that elapsed from the start of collections until the first mosquito of a given species was caught. If no mosquito of that species arrived during a particular collection, lag was set to 300 min (the full duration of collection). Lag to first approach differed significantly between heights for *Hg. janthinomys* (Kruskal–Wallis, DF = 2, χ^2^ = 6.4, *P* = 0.04), which arrived faster at 9 m than at 0 m (Wilcoxon Each Pair, *P* = 0.01), but did not differ between 0 m and 5 m or 5 m and 9 m (*P* > 0.1 for both comparisons). A linear regression showed that, across all heights, lag to first approach decreased significantly as *Hg. janthinomys* abundance increased (DF = 1, F = 52.1, *P* < 0.001), and the same pattern held when data from each height was analyzed separately (*P* < 0.005 for each analysis). As *Hg. janthinomys* abundance was higher at 9 m, this suggests that the difference among heights in lag to first approach is attributable to differences in abundance. When only considering collections in which least one *Hg. janthinomys* was caught, a multiple linear regression showed that lag to first approach did not differ by height but did differ by abundance (DF = 1, F = 16.7, *P* = 0.0001) and mean daytime temperature (DF = 1, F = 4.2, *P* = 0.04), with faster approaches occurring at higher abundance and higher temperature. Lag to first approach did not differ among heights for either *Ps. amazonica* or *Ps. albigenu*. Again, small sample sizes precluded analysis of *Sabethes* at species level. The mean time (+ / − 1 S.E.) to first approach for each species at each height is listed in Supplementary Table S4.

### Species occurrence

**Figure 5:**
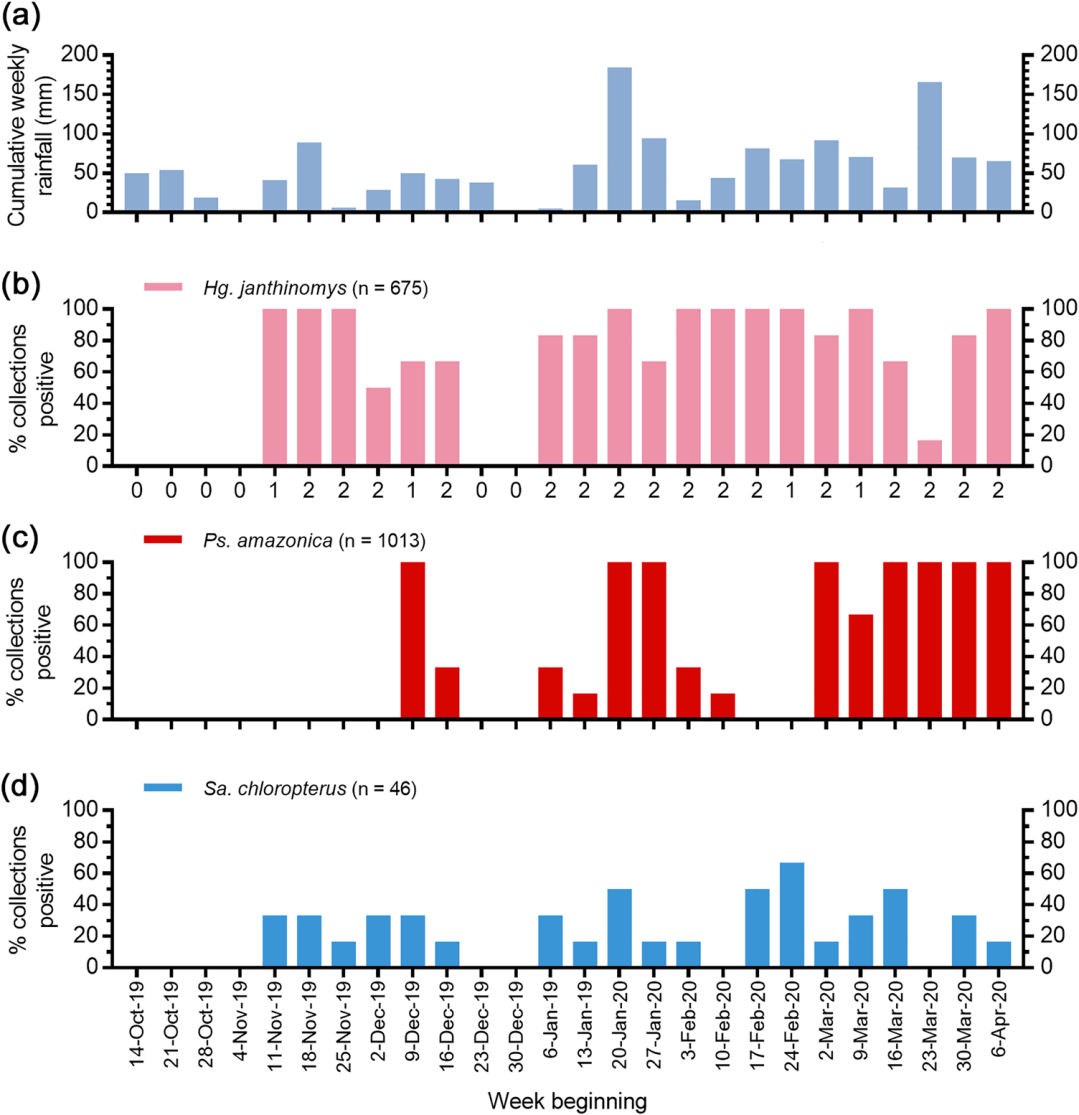
Rainfall and mosquito occurrence during the study period. (**a**) shows the cumulative weekly rainfall for the duration of the sampling period including four weeks prior to the start of collections. (**b**)**–**(**d**) show the weekly occurrence of *Hg. janthinomys*, *Ps. amazonica*, and *Sa. chloropterus*, while the number of mosquito sampling days per week is shown beneath the x-axis in panel (**b**). Occurrence was calculated as the percent of collections positive for a given species in a given week across all heights sampled, such that three collections were made each sampling day (one per each of three heights) and one to two days were sampled per week. Note that there was a sampling pause over the Christmas holiday (weeks staring 23 December 2019 and 30 December 2019).

Based on data summarized by date and height of collection, nominal logistic regressions revealed significant positive associations between several of the predictor variables and the occurrence of target species. Seven-day cumulative rainfall at a lag of 1 week (DF = 1, χ^2^ = 4.7, *P* = 0.03) was a significant positive predictor of *Hg. janthinomys* occurrence (Fig. 5), while height also had a marginal effect but was not significant in this analysis (DF = 2, χ^2^ = 5.1, P = 0.08). Rainfall at a lag of 1 week was associated with an increase in *Haemagogus* occurrence in our earlier study^15^. Date of collection (DF = 1, χ^2^ = 27.8, *P* < 0.0001), rainfall at a lag of one week (DF = 1, χ^2^ = 8.9, *P* = 0.003), and rainfall at a lag of four weeks (DF = 1, χ^2^ = 4.6, *P* = 0.03) were all significant predictors of *Ps. amazonica* occurrence (Fig. 5). Mean daytime temperature followed a similar but non-significant trend (DF = 1, χ^2^ = 3.3, *P* = 0.07) and height had no significant effect on the occurrence of this species. The association between *Ps. amazonica* occurrence and rainfall at lags of one and four weeks corresponded with our previous results^15^. Rainfall at a lag of one week (DF = 1, χ^2^ = 9.3, *P* = 0.002) was a significant predictor of *Ps. albigenu* occurrence, while height (DF = 2, χ^2^ = 5.0, *P* = 0.08) had a marginal but non-significant effect on this species which we had not previously analyzed^15^. Height was a significant predictor of *Sa. chloropterus* occurrence (DF = 2, χ^2^ = 14.5, *P* = 0.0007), but there was no effect of 7-day cumulative rainfall (Fig. 5). We had previously shown that *Sa. chloropterus* occurrence significantly increased with rainfall at a lag of 3 weeks^15^.

### Relationships between temperature and occurrence

**Figure 6:**
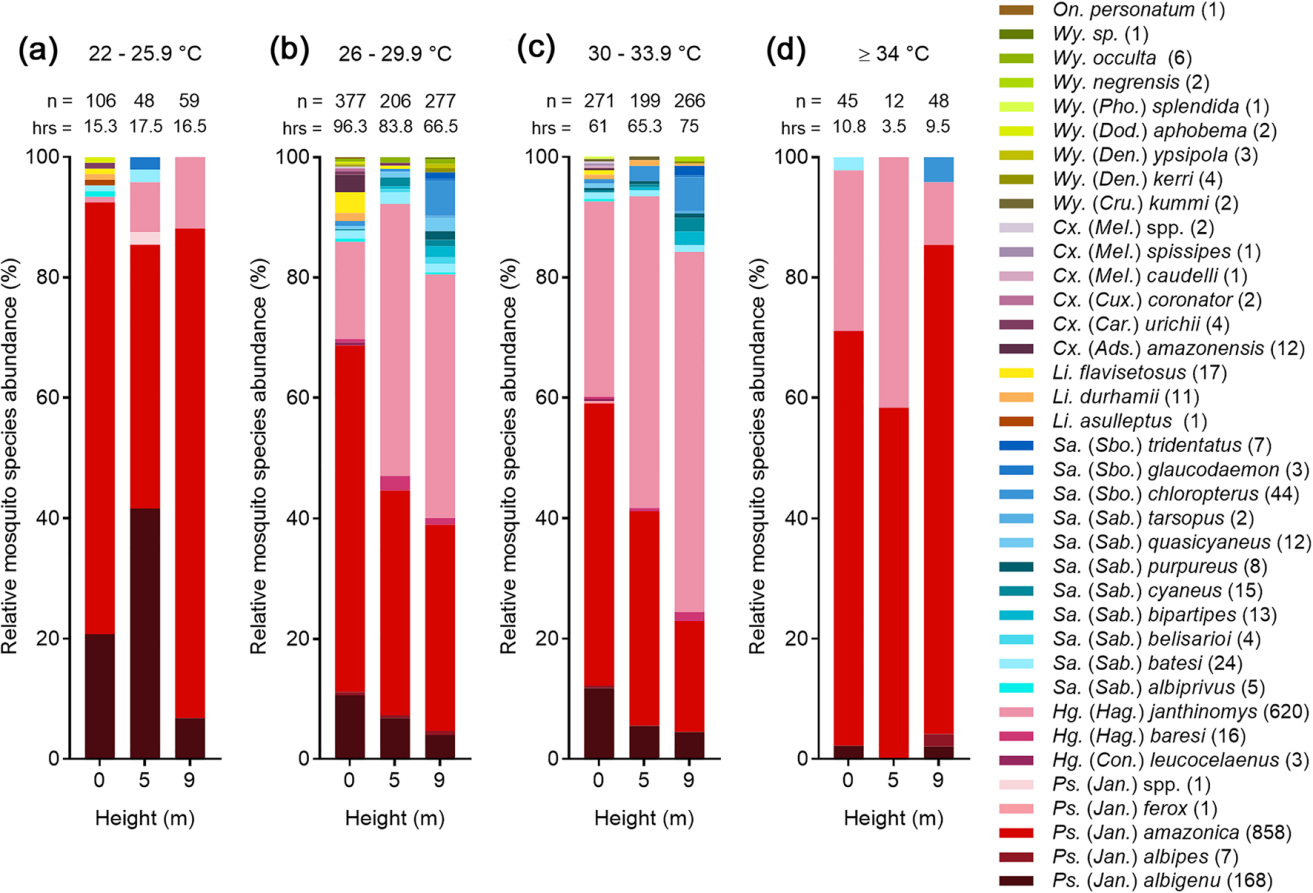
Relative mosquito species abundance by temperature and height for net collections. (**a**)**–**(**c**) show the relative abundance of species grouped in 4 °C temperature increments starting at 22 °C, while panel **d** shows species collected at 34 °C and above (maximum 38.6 °C). Number of mosquitoes (n =) collected and number of hours (hrs, rounded to one decimal place) that collections were made within each temperature and height class listed at top of bar; total number of individuals per species included in parentheses next to species name; genus and subgenus abbreviations follow Walter Reed Biosystematics Unit nomenclature^36^. Data were excluded for days where collections were made but iButtons malfunctioned.

BG-Sentinel trap collections had previously shown that *Haemagogus* and *Sabethes* species mosquitoes increased in occurrence with increasing temperature and decreasing relative humidity when analyzed at genus level^15^. In the current study, we found that these mosquitoes were mainly active between 26 °C and 33.9 °C when classified in ≈4 °C increments (Fig. 6), which were chosen to provide a balance between the range and sample size of each temperature class. Both *Ps. amazonica* and *Ps. albigenu* were more tolerant of lower temperatures (22–25.9 °C), while *Ps. amazonica* was the most abundant of the species collected at temperatures higher than 33.9 °C.

When analyzing relationships between species occurrence and microclimate using data summarized at 15-min time intervals by date and height, a nominal logistic regression revealed a significant interaction in the impact of height and temperature on the occurrence of *Hg. janthinomys* (DF = 1, χ^2^ = 4.4, *P* = 0.036). Subsequent nominal regressions analyzing the effect of temperature stratified by height showed a significant positive effect of temperature on occurrence at 0 m (DF = 1, χ^2^ = 13.9, *P* = 0.0002) and 5 m (DF = 1, χ^2^ = 17.0, P < 0.0001), but not at 9 m (DF = 1, χ^2^ = 2.0, *P* = 0.157). Interestingly, the mean temperature for the intervals in which *Hg. janthinomys* was collected was 29.9 °C at all three heights. Nominal logistic regressions showed no effect of temperature on the occurrence of *Ps. amazonica* (DF = 1, χ^2^ = 0.3, *P* = 0.58) but did reveal an effect on the occurrence of *Ps. albigenu* (DF = 1, χ^2^ = 7.3, *P* = 0.007), which preferred cooler than average conditions. There was no effect of temperature on the occurrence of either *Sa. chloropterus* (DF = 1, χ^2^ = 2.0, *P* = 0.15) or *Sabethes* when analyzed at genus level (DF = 1, χ^2^ = 0.3, P = 0.59), although sample sizes were small (n = 44 and n = 132, respectively).

### Community composition of mosquitoes in artificial and natural containers

**Figure 7:**
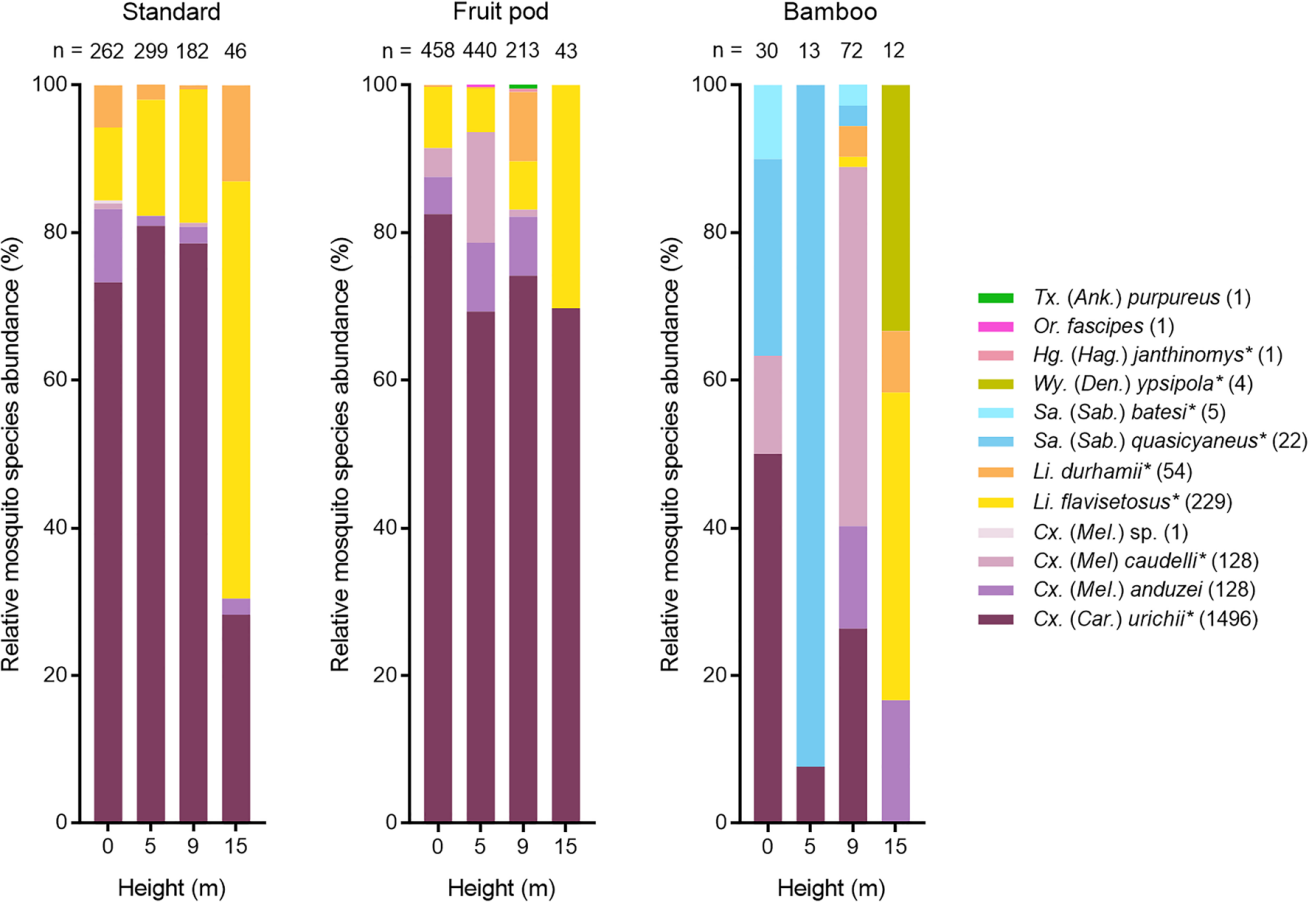
Relative mosquito species abundance by height and container type for standard, fruit pod, and bamboo ovitraps. Number of mosquitoes (n =) collected at each height listed above bar; number of individuals per species included in parentheses next to species name; *asterisk denotes species collected using both ovitraps and nets; genus and subgenus abbreviations follow Walter Reed Biosystematics Unit nomenclature^36^. Color scheme is consistent with previous figures.

A total of 2070 adult mosquitoes (50.9% female) representing seven genera and 11 species were reared from ovitraps, of which 99.9% were identified to species level (Fig. 7). In contrast to net collections, *Culex* was the most abundant genus (84.7% of the total catch), followed by *Limatus* (13.7%), *Sabethes* (1.3%), *Wyeomyia* (0.2%), *Haemagogus* (0.05%), *Orthopodomyia* (0.05%), and *Toxorhynchites* (0.05%). Collections in standard and fruit pod ovitraps were dominated by *Culex urichii* which comprised 75.2% of the catch in these containers, while *Limatus flavisetosus* (11.5%) was also relatively abundant. Mosquitoes reared from bamboo represented just 6.1% of the overall catch but included *Sa. quasicyaneus* (22 mosquitoes), *Sa. batesi* (5), and *Hg. janthinomys* (1), which were absent from other container types.

Mosquito communities in standard and fruit pod ovitraps overlapped substantially when analyzed using the Morisita index at species level (Supplementary Table S4) but overlap decreased when comparing these traps with bamboo. When comparing heights for the combined standard and fruit pod ovitraps, there was substantial overlap at 0, 5, and 9 m, but this decreased slightly when comparing each height with 15 m. When comparing heights for individual container types (Supplementary Table S5), the standard ovitrap followed a similar pattern, but the fruit pod showed substantial overlap among all heights sampled. Bamboo communities were quite dissimilar when comparing 0 m with elevated heights, and those at 15 m were distinct from all other heights sampled, but this was based on a small number of specimens.

## Discussion

The intersection of humans, mosquitoes, and monkeys in forests bordering urban environments creates risk for spillover and spillback of mosquito-borne viruses between urban and sylvatic cycles. We constructed a tower at the edge of a treefall gap to investigate relationships between microclimate and the vertical stratification of potential mosquito bridge vectors. Our results showed little difference in mean temperature and relative humidity across heights during the morning hours, but in the afternoon, it was hotter and less humid at 9 m than at ground level. This contrasted with the clear stratification of microclimate throughout the corresponding sampling period in our BG-Sentinel trap study^15^. Our findings at the tower mostly agreed with a study by Shuttleworth investigating the vertical stratification of microclimate at the Ducke reserve^40^. This showed that differences in temperature and humidity between heights gradually increased throughout the day and decreased at night, although temperature divergence began earlier in the morning than in our study. Others have also reported steep vertical gradients in temperature and humidity in the afternoon relative to the morning^41,42^. We observed higher mean temperatures at ground level than at 9 m during the morning collection hours and reasons for this may be explained by a breakdown in the vertical stratification of microclimate at forest edges, which has been shown to occur in temperate rainforest^43^.

We compared diurnally active, anthropophilic mosquito communities sampled with nets in this study with our previous BG-Sentinel trap collections^15^. In the current study, *Ps. amazonica*, *Hg. janthinomys*, and *Ps. albigenu* were the most abundant species, while *Sa. chloropterus* was poorly represented. In agreement with our earlier work^15^, the Morisita index showed substantial overlap in species composition between the sampled heights but indicated that elevated mosquito communities were more like each other than they were the ground level community. This was also reflected in the results of the principal components analysis. Other studies^44–46^ have shown similar changes in community composition with increasing height above the forest floor and found that species richness and mosquito abundance are generally higher at ground level than at the heights sampled in this study. We also found species richness to be greatest at 0 m, although overall abundance was similar between 0 and 9 m. The latter finding may be explained by our sampling methods which limited collections to one mosquito per minute, potentially underestimating abundance at ground level. This limitation was applicable to *Ps. amazonica* which was present in high relative abundance at ground level compared to elevated heights in BG-Sentinel traps but not in nets.

Our analyses of stratification showed that lag to first approach was closely negatively correlated with the abundance of our target species. The stratification in abundance of *Hg. janthinomys* sampled using nets was less prominent than in the BG-Sentinel trap study (Fig. 4). However, it differed between the morning and afternoon sampling periods, corresponding with differences in the stratification of microclimate (Fig. 3a and b). This species was only more abundant at 9 m than at ground level after midday, when temperatures were higher at elevated heights. Whereas several studies have indicated that *Hg. janthinomys* stratification shifts seasonally with changes in microclimate^18,28^, there is little known about diurnal shifts in this regard. Unfortunately, we were unable to investigate seasonal changes in stratification or community composition since our collections were truncated by the COVID-19 pandemic. We found no evidence of stratification in the abundance of *Ps. amazonica* or *Ps. albigenu* which were frequently caught at each height. While *Psorophora* species are known to be predominantly ground dwelling, they are often collected in the forest canopy^46,47^. In our study, the high relative abundance of both *Ps. amazonica* and *Hg. janthinomys* at each height sampled indicates a clear potential route for arbovirus transmission between the ground and forest canopy.

In contrast with our previous study^15^, the occurrence of *Hg. janthinomys* was not significantly stratified by height. There was also no effect of height on the occurrence of *Ps. amazonica* and *Ps. albigenu*. However, height did influence the occurrence of *Sa. chloropterus* in keeping with its acrodendrophilic behavior^15,47^. Several species showed significant associations with rainfall lag, an important predictor of mosquito emergence and vector-borne disease^48,49^. On this occasion, *Hg. janthinomys* occurrence increased with increasing rainfall at a lag of 1 week, an association that was previously reported for *Haemagogus* mosquitoes at genus level^15^. The occurrence of *Ps. amazonica* increased at lags of 1 and 4 weeks, concurrent with our previous results^15^. These findings support our earlier work and highlight reliable traits for modeling the emergence of both species. In addition, the occurrence of *Ps. albigenu* was associated with rainfall at a lag of 1 week which reflects the need for *Psorophora* species to develop quickly in transient breeding sites^50^. However, the association between *Sa. chloropterus* occurrence and rainfall at a lag of 3 weeks that we previously reported^15^ was not detected on this occasion, despite the sample size being slightly larger than in the earlier study. Since *Sa. chloropterus* develops slowly in large tree holes with small openings that minimize water loss^51,52^, its emergence may be less dependent on rainfall in the weeks immediately prior to collection than other species.

In agreement with our BG-Sentinel trap study^15^, we found that *Haemagogus* and *Sabethes* species were mainly active between ≈26 °C and 34 °C (Fig. 6). In addition, *Ps. amazonica* was more tolerant of more extreme temperatures while *Ps. albigenu* also tolerated cooler conditions. We previously suggested that relationships exist between the occurrence of *Haemagogus* and *Sabethes* mosquitoes and microclimate^15^ but had insufficient data to draw inference at species level. In this study, we showed that the mean temperature at time intervals in which *Hg. janthinomys* was collected was 29.9 °C at each height. This was greater than the mean daily temperature at ground level (29.0 °C) and shows that this species prefers warmer conditions. We also found that *Hg. janthinomys* occurrence increased with increasing temperature at 0 m and 5 m, but not at 9 m, where microclimate was more likely to be within its preferred range. Bates^18^ remarked that this species mainly occupied the lower canopy during his extensive investigations in Colombia, while Bates and Roca-Garcia^53^ showed that it was more tolerant than other local mosquitoes to laboratory temperatures of 30 °C. These findings agree with our evidence that *Hg. janthinomys* abundance was not significantly stratified in the morning when temperatures were similar at each height. They may also explain the differences in relative abundance of *Hg. janthinomys* collected at ground level in nets and in traps (Fig. 4). Whereas net collections were often made in direct sunlight, BG-Sentinel traps were mainly hung beneath the forest canopy where microclimate was cooler and more humid than in the current study^15^. This was especially true at ground level where the mean temperature was never higher than 27.2 °C and *Hg. janthinomys* was rarely caught. In addition to microclimate influencing stratification, a recent study of the sylvan malaria vector, *Anopheles cruzii,* suggests that mosquitoes present at ground level may have a different genetic structure to those found in the forest canopy, and that anthropogenic habitat fragmentation may drive the vertical dispersal of particular genotypes^54^.

In addition to collecting diurnally active, anthropophilic host-seeking mosquitoes, the tower also enabled us to conveniently sample certain container-breeding species. The composition of mosquitoes collected with ovitraps was strikingly different to those sampled with nets and BG-Sentinel traps. Despite rearing over 2000 adults from the three container types, the abundance of target species was too low to investigate vertical stratification. The predominant mosquito in standard and fruit pod ovitraps was *Cx. urichii*, a diurnal species^55^ recently found in high abundance near forest edges at a rural settlement north of Manaus^56^. However, there is little evidence to suggest that *Cx. urichii* is attracted to humans^55,57^. The bamboo community was quite distinct from the other ovitraps and was the only container type to yield *Sabethes* mosquitoes. While bamboo ovitraps are useful for collecting certain *Haemagogus* and *Sabethes* species^25,56^, Galindo et al.^47,51,58^ have shown that the abundance of *Hg. janthinomys* and *Sa. chloropterus* sampled with this method is not always commensurate with the abundance of adult populations. We only reared a single *Hg. janthinomys* specimen from bamboo despite this species being common at our study site, although our methods differed slightly from others that have proven more successful for collecting this species^59,60^.

Our study primarily focused on the population of diurnally active, anthropophilic mosquitoes most likely to effect spillover and spillback in rainforest bordering Manaus. While we identified several potential routes of transmission for mosquito-borne viruses between the ground and forest canopy, there is scope to further investigate crepuscular and nocturnal species not detected during the current study.

## Supplementary Information

Supplementary Information 1.

Supplementary Information 2.

Supplementary Information 3.

## Data Availability

All data generated or analyzed during this study are included in this published article (and its Supplementary Information files).

## References

[1] Hanley KA (2013). Fever versus fever: the role of host and vector susceptibility and interspecific competition in shaping the current and future distributions of the sylvatic cycles of dengue virus and yellow fever virus. Infect. Genet. Evol..

[2] Bryant JE, Holmes EC, Barrett AD (2007). Out of Africa: a molecular perspective on the introduction of yellow fever virus into the Americas. PLoS Pathog..

[3] Sacchetto L, Drumond BP, Han BA, Nogueira ML, Vasilakis N (2020). Re-emergence of yellow fever in the neotropics - quo vadis?. Emerg. Top. Life Sci..

[4] Escosteguy CC (2019). Yellow fever: profile of cases and factors associated with death in a hospital in the State of Rio de Janeiro, 2017–2018. Rev. Saude Publica.

[5] Hendy A (2020). Into the woods: Changes in mosquito community composition and presence of key vectors at increasing distances from the urban edge in urban forest parks in Manaus, Brazil. Acta Trop..

[6] Chippaux, J.-P. & Chippaux, A. Yellow fever in Africa and the Americas: a historical and epidemiological perspective. *J. Venom. Anim. Toxins Incl. Trop. Dis.* **24**, 20, doi:10.1186/s40409-018-0162-y (2018). 10.1186/s40409-018-0162-y PMC6109282 30158957

[7] World Health Organization. *Yellow fever, Brazil*, https://www.who.int/csr/don/18-april-2019-yellow-fever-brazil/en/ (2019).

[8] Silva NIO (2020). Recent sylvatic yellow fever virus transmission in Brazil: the news from an old disease. Virol J..

[9] Cunha MS (2020). Possible non-sylvatic transmission of yellow fever between non-human primates in São Paulo city, Brazil, 2017–2018. Sci. Rep..

[10] Callender DM (2019). Management and control of yellow fever virus: Brazilian outbreak January–April, 2018. Glob Public Health.

[11] Guth S, Hanley KA, Althouse BM, Boots M (2020). Ecological processes underlying the emergence of novel enzootic cycles: arboviruses in the neotropics as a case study. PLoS Negl. Trop. Dis..

[12] Lowe R (2020). Emerging arboviruses in the urbanized Amazon rainforest. BMJ.

[13] Althouse BM (2016). Potential for Zika virus to establish a sylvatic transmission cycle in the Americas. PLoS Negl. Trop. Dis..

[14] Lourenço-de-Oliveira R, Failloux AB (2017). High risk for chikungunya virus to initiate an enzootic sylvatic cycle in the tropical Americas. PLoS Negl. Trop. Dis..

[15] Hendy A (2020). The vertical stratification of potential bridge vectors of mosquito-borne viruses in a central Amazonian forest bordering Manaus, Brazil. Sci. Rep..

[16] Trapido H, Galindo P (1957). Mosquitoes associated with sylvan yellow fever near Almirante, Panama. Am. J. Trop. Med. Hyg..

[17] Garnham PC, Harper JO, Highton RB (1946). The mosquitos of the Kaimosi Forest, Kenya Colony, with special reference to yellow fever. Bull. Entomol. Res..

[18] Bates M (1944). Observations on the distribution of diurnal mosquitoes in a tropical forest. Ecology.

[19] Biogents AG. *The BG-Sentinel: Biogents' mosquito trap for researchers*, https://www.bg-sentinel.com/ (2020).

[20] Dégallier, N., Sá Filho, G. C., Silva, O. V. & Travassos da Rosa, A. P. A. Comportamento de pouso sobre partes do corpo humano, em mosquitos da floresta amazonica (Diptera: Culicidae). *Bol. Mus. Para. Emilio Goeldi. Nova Série, Zoologia* **6**, 97–108, (1990).

[21] Forattini, O. P., Kakitani, I., Massad, E. & Marucci, D. Studies on mosquitoes (Diptera: Culicidae) and anthropic environment. 4 - Survey of resting adults and synanthropic behaviour in south-eastern Brazil. *Rev. Saude Publica* **27**, 398–411, doi:10.1590/s0034-89101993000600002 (1993). 10.1590/s0034-89101993000600002 7997810

[22] Silver, J. B. & Service, M. W. *Mosquito ecology: Field sampling methods*. 3rd edn, pp. 1494 (Springer, Dordrecht, the Netherlands, 2008).

[23] Obenauer PJ, Kaufman PE, Kline DL, Allan SA (2010). Detection of and monitoring for *Aedes albopictus* (Diptera: Culicidae) in suburban and sylvatic habitats in north central Florida using four sampling techniques. Environ. Entomol..

[24] Mangudo C, Aparicio JP, Rossi GC, Gleiser RM (2018). Tree hole mosquito species composition and relative abundances differ between urban and adjacent forest habitats in northwestern Argentina. Bull. Entomol. Res..

[25] Santos M (2020). Implementation of bamboo and monkey-pot traps for the sampling cavity-breeding mosquitoes in Darién, Panama. Acta Trop..

[26] Young KI (2017). Abundance and distribution of sylvatic dengue virus vectors in three different land cover types in Sarawak Malaysian, Borneo. Parasit. Vectors..

[27] Mayi MPA (2020). Habitat and seasonality affect mosquito community composition in the West Region of Cameroon. Insects.

[28] Galindo P, Trapido H, Carpenter SJ, Blanton FS (1956). The abundance cycles of arboreal mosquitoes during six years at a sylvan yellow fever locality in Panama. Ann. Entomol. Soc. Am..

[29] FVS/AM. Boletim Epidemiológico Arboviroses. Report No. ANO 2020 - Nº2, pp. 8 (Fundação de Vigilância em Saúde do Amazonas, Manaus, Brazil, 2020).

[30] Ministério da Saúde. Emergência epidemiológica de febre amarela no Brasil, no período de dezembro de 2016 a julho de 2017. Report No. N° 28 - 2017, pp. 22 (Brasília, 2017).

[31] Oliveira, M. L. d., Baccaro, F. B., Braga-Neto, R. & Magnusson, W. E. (eds). *Reserva Ducke: A bioversidade Amazônica através de uma grade*. pp. 166 (Instituto Nacional de Pesquisas da Amazônia, Manaus, 2011).

[32] Mittermeier RA, van Roosmalen MG (1981). Preliminary observations on habitat utilization and diet in eight Surinam monkeys. Folia Primatol. (Basel).

[33] Egler SG (1992). Feeding ecology of *Saguinus bicolor bicolor* (Callitrichidae: Primates) in a relict forest in Manaus, Brazilian Amazonia. Folia Primatol. (Basel).

[34] Lenz BB, Jack KM, Spironello WR (2014). Edge effects in the primate community of the biological dynamics of Forest Fragments Project, Amazonas, Brazil. Am. J. Phys. Anthropol..

[35] Trapido H, Galindo P, Carpenter SJ (1955). A survey of forest mosquitoes in relation to sylvan yellow fever in the Panama Isthmian area. Am. J. Trop. Med. Hyg..

[36] Walter Reed Biosystematics Unit. *Systematic catalog of Culicidae*, http://mosquitocatalog.org/default.aspx (2020).

[37] Morisita M (1962). Iσ-Index, a measure of dispersion of individuals. Res. Popul. Ecol. (Kyoto).

[38] Hammer Ø, Harper DAT, Ryan PD (2001). PAST: paleontological statistics software package for education and data analysis. Palaeontol. Electronica.

[39] JMP v14 (SAS Institute Inc, Cary, NC, USA, 2018).

[40] Shuttleworth WJ (1985). Daily variations of temperature and humidity within and above Amazonian forest. Weather.

[41] Barker, M. G. Vertical profiles in a Brunei rain forest: I. Microclimate associated with a canopy tree. *J Trop For Sci* **8**, 505–519 (1996).

[42] Madigosky SR, Vatnick I (2000). Microclimatic characteristics of a primary tropical Amazonian rain forest, Aceer, Iquitos, Peru. Selbyana.

[43] Didham RK, Ewers RM (2014). Edge effects disrupt vertical stratification of microclimate in a temperate forest canopy. Pac. Sci..

[44] Confalonieri UE, Costa Neto C (2012). Diversity of mosquito vectors (Diptera: Culicidae) in Caxiuanã, Pará, Brazil. Interdiscip. Perspect. Infect. Dis..

[45] Brant HL (2016). Vertical stratification of adult mosquitoes (Diptera: Culicidae) within a tropical rainforest in Sabah, Malaysia. Malar. J..

[46] Pereira-Silva JW (2021). Distribution and diversity of mosquitoes and Oropouche-like virus infection rates in an Amazonian rural settlement. PLoS ONE.

[47] Galindo P, Trapido H, Carpenter SJ (1950). Observations on diurnal forest mosquitoes in relation to sylvan yellow fever in Panama. Am. J. Trop. Med. Hyg..

[48] Anyamba A (2012). Climate teleconnections and recent patterns of human and animal disease outbreaks. PLoS Negl. Trop. Dis..

[49] Tian HY (2015). How environmental conditions impact mosquito ecology and Japanese encephalitis: an eco-epidemiological approach. Environ. Int..

[50] Bates M (1945). Observations on climate and seasonal distribution of mosquitoes in eastern Colombia. J. Anim. Ecol..

[51] Galindo P, Carpenter SJ, Trapido H (1951). Ecological observations on forest mosquitoes of an endemic yellow fever area in Panama. Am. J. Trop. Med. Hyg..

[52] Galindo P (1958). Bionomics of *Sabethes chloropterus* Humboldt, a vector of sylvan yellow fever in Middle America. Am. J. Trop. Med. Hyg..

[53] Bates, M. & Roca-Garcia, M. Laboratory studies of the *Saimiri*-*Haemagogus* cycle of jungle yellow fever. *Am. J. Trop. Med. Hyg.* **s1–25**, 203–216, doi:10.4269/ajtmh.1945.s1-25.203 (1945).

[54] Multini, L. C., de Souza, A. L. d. S., Marrelli, M. T. & Wilke, A. B. B. The influence of anthropogenic habitat fragmentation on the genetic structure and diversity of the malaria vector *Anopheles cruzii* (Diptera: Culicidae). *Sci. Rep.* **10**, 18018, doi:10.1038/s41598-020-74152-3 (2020). 10.1038/s41598-020-74152-3 PMC7581522 33093465

[55] Valencia, J. D. Mosquito studies (Diptera, Culicidae) XXXI. A revision of the subgenus *Carrollia* of *Culex*. *Contrib. Am. Entomol. Inst.* **9**, 1–134, (1973).

[56] Almeida JF, Belchior HCM, Ríos-Velásquez CM, Pessoa FAC (2020). Diversity of mosquitoes (Diptera: Culicidae) collected in different types of larvitraps in an Amazon rural settlement. PLoS ONE.

[57] Chadee DD, Tikasingh ES (1986). The eggs of *Culex* (*Carrollia*) *urichii* (Coquillett) (Diptera: Culicidae) in Trinidad. Mosquito Syst..

[58] Galindo P, Carpenter SJ, Trapido H (1955). A contribution to the ecology and biology of tree hole breeding mosquitoes of Panama. Ann. Entomol. Soc. Am..

[59] Alencar J, de Almeida HM, Marcondes CB, Guimarães AÉ (2008). Effect of multiple immersions on eggs and development of immature forms of *Haemagogus janthinomys* from south-eastern Brazil (Diptera: Culicidae). Entomol. News.

[60] Silva SOF (2021). Oviposition behavior of wild yellow fever vector mosquitoes (Diptera: Culicidae) in an Atlantic Forest fragment, Rio de Janeiro state, Brazil. Sci. Rep..

