# Supplementary material for: Microclimate and the vertical stratification of potential bridge vectors of mosquito‑borne viruses captured by nets and ovitraps in a central Amazonian forest bordering Manaus, Brazil
Source: Sci Rep. 2021 Oct 26;11:21129. doi: 10.1038/s41598-021-00514-0 (PMC8548557; doi:10.1038/s41598-021-00514-0)
Supplement: Supplementary file 3 — Supplementary Information 3. [file 41598_2021_514_MOESM3_ESM.docx]

Supplementary Information

Microclimate and the vertical stratification of potential bridge vectors of mosquito‑borne viruses captured by nets and ovitraps in a central Amazonian forest bordering Manaus, Brazil

Adam Hendy, Danielle Valério, Nelson Ferreira Fé, Eduardo Hernandez-Acosta, Claudia Mendonça, Eloane Andrade, Igor Pedrosa, Edson Rodrigues Costa, José Tenaçol Andes Júnior, Flamarion Prado Assunção, Bárbara Aparecida Chaves, Vera Margarete Scarpassa, Marcelo Gordo, Michaela Buenemann, Marcus Vinícius Guimarães de Lacerda, Kathryn A. Hanley, Nikos Vasilakis

**Table S3.** Mean lag to first approach in minutes +/- 1 standard error (S.E.) for each species analyzed at each height.

|  | *Hg. janthinomys* | | *Ps. amazonica* | | *Ps. albigenu* | |
| --- | --- | --- | --- | --- | --- | --- |
| **Height** | **Lag (mins)** | **± 1 S.E.** | **Lag (mins)** | **± 1 S.E.** | **Lag (mins)** | **± 1 S.E.** |
| 0 m | 147.2 | 19.6 | 152.7 | 23.0 | 195.4 | 20.5 |
| 5 m | 125.6 | 18.7 | 187.3 | 22.2 | 244.5 | 16.6 |
| 9 m | 85.4 | 16.0 | 180.7 | 21.2 | 223.4 | 16.1 |

**Table S4.** Morisita overlap index by ovitrap type, and by height for combined standard and fruit pod ovitraps, based on specimens identified to species level.

| **Ovitrap** | Standard | Fruit pod | Bamboo | **Height** | 0 m | 5 m | 9 m | 15 m |
| --- | --- | --- | --- | --- | --- | --- | --- | --- |
| Standard | 1 |  |  | 0 m | 1 |  |  |  |
| Fruit pod | 0.988 | 1 |  | 5 m | 0.994 | 1 |  |  |
| Bamboo | 0.547 | 0.603 | 1 | 9 m | 0.997 | 0.992 | 1 |  |
|  |  |  |  | 15 m | 0.791 | 0.805 | 0.824 | 1 |

**Table S5.** Morisita index by height for individual container types. The single mosquito that was not identified to species (*Cx.* (*Mel.*) sp.) was excluded from the analysis.

Data for the **standard** ovitrap only.

| **Height** | 0 m | 5 m | 10 m | 15 m |
| --- | --- | --- | --- | --- |
| 0 m | 1 |  |  |  |
| 5 m | 0.986 | 1 |  |  |
| 10 m | 0.985 | 0.999 | 1 |  |
| 15 m | 0.558 | 0.584 | 0.610 | 1 |

Data for the **fruit pod** (gourd) ovitrap only.

| **Height** | 0 m | 5 m | 10 m | 15 m |
| --- | --- | --- | --- | --- |
| 0 m | 1 |  |  |  |
| 5 m | 0.973 | 1 |  |  |
| 10 m | 0.986 | 0.972 | 1 |  |
| 15 m | 0.946 | 0.917 | 0.936 | 1 |

Data for the **bamboo** ovitrap only.

| **Height** | 0 m | 5 m | 10 m | 15 m |
| --- | --- | --- | --- | --- |
| 0 m | 1 |  |  |  |
| 5 m | 0.472 | 1 |  |  |
| 10 m | 0.611 | 0.077 | 1 |  |
| 15 m | 0 | 0 | 0.1 | 1 |
